# Revisiting we are MLA: an exploration of member engagement and commitment with the Medical Library Association's caucuses

**DOI:** 10.5195/jmla.2026.2183

**Published:** 2026-01-01

**Authors:** Rachel Whitney, Kimberly R. Powell, Michelle Keba Knecht, Rachel Lane Walden

**Affiliations:** 1 whitnera@musc.edu, Research and Education Informationist, MUSC Libraries, Medical University of South Carolina, SC; 2 krp0069@auburn.edu, Health and Life Sciences Librarian, Auburn University Libraries, Auburn University, Auburn, AL; 3 kebam@health.fau.edu, Senior Medical Librarian and Head of the Medical and Health Sciences Collection and User Services Department, Charles E. Schmidt College of Medicine, Florida Atlantic University, Boca Raton, FL; 4 rachel.l.walden@vanderbilt.edu, Assistant Director for Research and Education Services, Annette and Irwin Eskind Family Biomedical Library and Learning Center, Vanderbilt University, Nashville, TN

**Keywords:** Medical Library Association, Health Science Librarians, Community Engagement, Organizational Commitment, Professional organizations, organizational change, library association management

## Abstract

In 2019 the Medical Library Association (MLA) transitioned to a community structure composed of caucuses. Four years after the transition, the 2023-2024 MLA Rising Stars cohort was asked to investigate how the caucuses were currently functioning and any challenges to their sustainability. This Special Paper will describe the study conducted by the Rising Stars cohort, and its research findings. Preliminary recommendations include greater standardization of annual reporting, additional guidance and discussion forums for caucus leadership, and an increase in events focused on professional development, networking, and information sharing such as those held during Experience MLA.

## INTRODUCTION

The Medical Library Association (MLA) offers an annual leadership development program called the Rising Stars [1]. Consisting of a cohort of four MLA members, participants attend monthly meetings on a variety of leadership topics and are paired with a mentor. Each year the cohort is tasked with completing a group project which relates to current MLA initiatives. The 2023-2024 Rising Stars cohort was asked to investigate the MLA caucuses including challenges to sustainability and current functions. The overall goal of the project was to create a list of recommendations for leadership recruitment and member engagement with MLA caucuses.

### Transition to Caucuses

To provide context for the current MLA caucus structure, prior to 2019, MLA had a two-tiered community structure composed of sections and special interest groups (SIGs). Members had to pay to join sections, and each section managed its own budget. Sections had a required leadership and reporting structure and participated in MLA's Community Council. SIGs were free to join, had minimal leadership, and were not required to report their activities to MLA, nor invited to participate in Community Council. MLA's Community Council served as the governing body for section leadership to advise the MLA Board of Directors and facilitate collaboration between groups. Following the transition, MLA Community Council continues as a representative body and offers a forum for collaboration among caucus leaders. The transition to caucuses was implemented in 2019 to increase member engagement, create more inclusive community structures, and reduce administrative overhead [2,3]. Prior to the transition, the 2019 MLA annual report listed twenty-six SIGs and twenty-one active sections. Based on the 2020 annual report, thirty-seven of those groups made the transition to become a caucus. As of the 2023 report, there were forty-two active caucuses, with eight having formed since the 2020 annual report. Following the transition to caucuses, two groups later elected to merge into other caucuses and one newly created caucus also disbanded within the four-year time frame.

### Research Objective

The primary objective of this study was to determine ways to increase caucus engagement and sustainability by answering the question: “What factors influence member engagement and commitment to an MLA caucus?”

## LITERATURE REVIEW

Four studies have examined membership engagement within MLA [3–6]. Two of these [3, 4] were previous Rising Star projects looking at aspects of MLA community engagement, though one predated the 2019 transition to the current caucus group structure [3]. The study conducted by the 2016-2017 cohort investigated ways to make sections and SIGs more effective and meaningful to MLA members [3]. The We are MLA study conducted by the 2019-2020 cohort sought to ascertain transition perceptions and change management feedback as groups moved to the current caucus structure by interviewing MLA members who held leadership roles in MLA committees, sections, or SIGs during the transition [4]. Both the 2016-2017 and 2019-2020 cohorts noted specific member concerns around organizational communication, change leadership, and time and financial burdens to member engagement. Specific barriers called out a lack of guidance, data tracking, or clear objectives when participating in community leadership. Similarly, a lack of member awareness of community activities and efforts as well as difficulty navigating the website were reported in both studies [3, 4].

The other two studies [5,6] did not examine section or caucus engagement directly, but their surveys provide important benchmarking data for membership demographics. Reporting on voter engagement survey data from 2017, Shedlock and McQuillen found that 76% of respondent members belonged to MLA Sections from a total number of 676 survey participants [5]. Reporting on the results of the 2019 survey from the Diversity and Inclusion Task Force, Pionke found that 69% of the 918 respondents either agreed or strongly agreed that they had found an MLA community or group in which to belong, though only 59% reported a sense of belonging within the larger organization [6].

Outside of MLA, two additional studies investigated membership engagement within library professional organizations [7, 8]. Publishing in 2014, Henczel noted the decline of membership in national library associations, citing in part increased member costs, growing demands of professional roles, perceived value, and irrelevancies [7]. Fifty-two semi-structured interviews were conducted across four national library societies: Australian Library and Information Association (ALIA), Library and Information Association of New Zealand (LIANZA), American Library Association (ALA), and Chartered Institute of Library and Information Professionals (CILIP) in the United Kingdom. Themes from participants highlighted the perceived benefits of professional membership in national library organizations as skills development, advocacy and professional standards, and providing a sense of belonging and professional community. However, respondents expressed concerns around the organizations’ disconnect with recruiting and engagement with library schools and training programs, as well as with staying relevant with greater and evolving workplace demands.

Echoing concerns regarding membership decline among librarian professional organizations and a questioning of the value of these organizations, Garrison and Cramer surveyed 140 self-identified U.S. business librarians in 2019 for the defining characteristics of successful library organizations [8]. Respondents reported ‘continued relevancy’ and ‘great programming’ as their top criteria, with on-going training opportunities, good leadership, and reasonable membership fees as additional considerations. When asked to reflect on their disappointment with library professional groups, respondents selected poor communication from the organization to its members as the top reason.

Library organizations are not alone in reflecting on membership and engagement. Within the broader body of literature on volunteer engagement and organizational commitment, research has shown that member commitment is driven primarily by volunteer satisfaction and needs fulfillment. For example, one study of 245 volunteers across 5 organizations highlighted that volunteer satisfaction was a key variable for members' commitment and intention to remain in an organization [9]. Elements used to define volunteer satisfaction included alignment with personal values, professional training and career growth opportunities, and the perceived clarity, utility, and efficiency of task objectives. Another study of over 13,000 members from 18 professional organizations, found positive correlations between the perceived value of the organization and tangible organizational support with increased volunteerism and donation activities, most notably among junior members [10].

Together these findings helped build a framework for understanding the important aspects of MLA caucuses and methods for measuring participants’ perceptions of value and belonging. Previous MLA membership surveys provided important baselines for participant demographics and engagement structures as well as persistent barriers to members' sense of commitment and satisfaction within the organization [5,6], but did not investigate how those factors have changed in the years following the transition to caucuses. The 2023-2024 Rising Star Cohort thus adapted the assigned leadership development topic into a specific investigation of “What factors influence member engagement and commitment to an MLA Caucus?”

## METHODS

Following the completion of the literature search, the authors identified two key sources of data to inform their findings. First, they identified baselines of participation and perceived barriers to caucus engagement by surveying the MLA membership. Second, they reviewed the engagement opportunities offered by MLA caucuses through an analysis of the caucuses' annual reporting.

### Survey

The authors conducted an anonymous survey of all MLA members during November and December of 2023. When the survey was distributed, there were 2,497 MLA members. Members received the survey via email, and the authors also shared a survey link on the MedLib-L listserv. Because the survey focused on internal organizational practices and perceptions aimed at quality improvement within the Medical Library Association, it was ruled exempt by the Institutional Review Board at Florida Atlantic University (IRB2309125) and deemed quality improvement and therefore not subject to review by the other authors’ institutions.

The twenty-question survey was hosted in RedCap and asked how and why members engaged with caucuses, their commitment as measured through perceived sense of belonging, barriers to getting involved with MLA caucuses, and basic demographic information. Using information gleaned from the literature review, the authors drafted survey questions, had them reviewed by the 2023–2024 Rising Stars Program Directors and Mentors as well as MLA staff, and piloted the survey with MLA members. With permission, the authors replicated many of the demographic questions from the survey created by Pionke to validate the cross-section of member responses to our own survey [6]. Due to the limited timeframe of the Rising Stars program, open-ended questions were not included in the survey. The entire survey instrument can be found in Appendix A.

### Thematic Analysis

The authors conducted a thematic content analysis of annual caucus reports from June 2019 - May 2023. The goal of the thematic content analysis was to determine the types of activities being reported by each caucus. The June 2019 - May 2020 reporting year marked the first annual report following the transition to caucuses and the June 2022 - May 2023 was the most recent annual report available at the time of this study. Every caucus submitted an annual report each year, though some missed the reporting deadline and were only available as supplemental documents.

Through the thematic content analysis, the authors produced a list of activity types that could be used to categorize and track the events and activities documented by each caucus in their annual report. The activity type categories were then used to create a caucus activities matrix in Excel with a row for each caucus and a column for each activity type. After pilot testing the matrix with the most recent reporting year, the authors narrowed the activity type categories to a total of ten, covering the full range of reported efforts included in the annual reports. A full list of these categories can be found in Appendix B.

Each report was read and documented on the matrix by two independent reviewers, with any disagreements settled by consensus of all four authors. Consensus was vital to this process because while there are specific sections required in the annual reports, there didn’t seem to be consistency or guidelines about what needed to be included in each section and with what level of detail. Within the matrix, the authors only noted the type of caucus activities reported by each group rather than the frequency of the designated activity. For example, though a caucus may have reported two standing committees and one working group this would all have been noted once in the matrix under the single activity type “Working Groups, Task Force and/or Committees.” Similarly, each reported activity was noted under only one activity type. For example, if a caucus hosted a discussion event that focused on networking this was noted once in the matrix under the “Networking Opportunities” activity type and was not noted simultaneously under the “Webinars and/or Discussions” activity type.

## RESULTS

### Caucuses Overview

A total of 44 caucuses completed annual activity reports from 2019-2023. Three caucuses disbanded or merged during this time period, and 4 caucuses were created. By member size, caucuses ranged from 59 members to 800, with a median membership of 227, as of October 2023. Groups that were created since 2019 had a median membership of 252 as of October 2023, while those that have disbanded or merged had a median membership of 127 at their final counts. Appendix C provides a full overview of each caucus and member size.

### Demographics from Survey

The survey was completed by 317 people, for an estimated 13% response rate. Not all respondents answered every question. Nearly all respondents (97%, n=305/315) reported that they currently live, work, or study in the United States. When asked about their work setting, 62% (n=196/317) of respondents reported working in an academic environment, including institutions offering 2-year, 4-year, graduate, or postgraduate programs. This was followed by 24% (n=75/317) of respondents working in a hospital or healthcare system. When asked to indicate their racial or ethnic identity, most respondents (74%, n=228/310) identified as White or Caucasian. Other racial or ethnic groups represented include respondents who identified as Black or African American (6%, n=18/310), Multiracial (5%, n=16/310), Hispanic/Latinx (5%, n=14/310), and Asian or Asian American (3%, n=9/310). Most respondents were over the age of 40 (73%, n=230/315), followed by ages 30-39 (19%, n=61/315). When asked if they considered themselves solo librarians, 14% (n=42/303) indicated that they currently work as solo librarians, while an additional 18% (n=54/303) reported that they are not currently solo librarians but had previously worked as one. The complete demographic responses can be seen in Appendix D.

### MLA Membership Information from Survey

Most respondents (98%, n=308/313) indicated that they were current members of the Medical Library Association (MLA) at the time of survey completion. Regarding the duration of their MLA membership, the largest group (21%, n=67/315) had been members for 5–9 years, followed by 18% (n=56/315) with 10–14 years of membership. Both those who had been members for 15–19 years and those with 25 or more years of membership each account for 15% (n=46/315).

When asked if their employer pays for their annual MLA membership, 39% (n=124/317) indicated that their employer does not pay for their membership. Conversely, 32% (n=100/317) reported that their employer fully covers the membership fee outside of any professional development funds, and 17% (n=55/317) noted that their employer pays the full membership fee if they choose to allocate professional development funds for it.

### Leadership Information from Survey

In terms of leadership roles in MLA, 33% (n=105/314) indicated that they currently hold a leadership position in an MLA group or community, such as a caucus, committee, or jury. An additional 18% (n=57/314) reported that they previously held a leadership position between 2019 and 2023. However, many respondents (48%, n=152/314) indicated that they had not held any leadership position during this period.

Regarding respondents' current or past leadership roles, of the 162 respondents who currently or previously held a leadership position since 2019, the majority served in caucuses (62%, n=100), followed by juries (31%, n=51), and standing committees (29%, n=47). Other significant leadership areas included domain hubs (14%, n=23), task forces (10%, n=16), and editorial boards (6%, n=9). Smaller numbers held positions in the Chapter Council (4%, n=7) and Community Council (2%, n=4). A few participated in the Rising Stars Program (1%, n=2), while less than 1% served as MLA Fellows (n=1), in the Research Training Institute (n=1), or as Parliamentarians (n=1). Lastly, 6% had been members of the MLA Board of Directors (n=10).

### Engagement and Sense of Belonging in Caucuses from Survey

**Figure 1:**
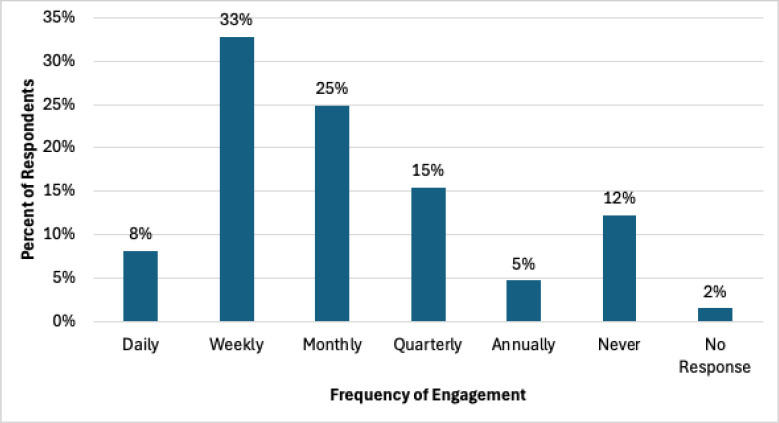
Frequency of engagement.

While there are currently over forty caucuses for members to join, 33% (n=104/315) of respondents reported they were members in 3-5 caucuses, followed by 21% (n=65/315) in 1-2 caucuses, 18% (n=56/315) in 9-19 caucuses, and 15% (n=47/315) in 6-8 caucuses. 10% (n=31/315) of respondents were not a member in any caucus and 4% (n=12/315) were members in over 20 caucuses. Figure 1 displays how often respondents reported engaging with a MLA caucus ranging from daily to annually, regardless of the number of caucuses joined.

Table 1 displays the relationship between the number of caucuses respondents joined and the frequency of their engagement with those caucuses. Those in 3-5 caucuses and those in over 20 caucuses were most likely to engage weekly. However, respondents in 6-8 caucuses and 9-19 caucuses were about as likely to engage weekly as they were to engage monthly, and respondents in 1-2 caucuses were slightly more likely to engage monthly, closely followed by weekly and then quarterly.

**Table 1:** Number of caucuses joined and frequency of engagement.

	Daily	Weekly	Monthly	Quarterly	Annually	Never
**None**	0	0	0	5	2	23
**1-2**	4	15	18	14	6	6
**3-5**	9	43	25	18	4	5
**6-8**	5	18	17	3	2	2
**9-19**	7	20	17	8	1	1
**20+**	1	8	2	1	0	0

**Figure 2:**
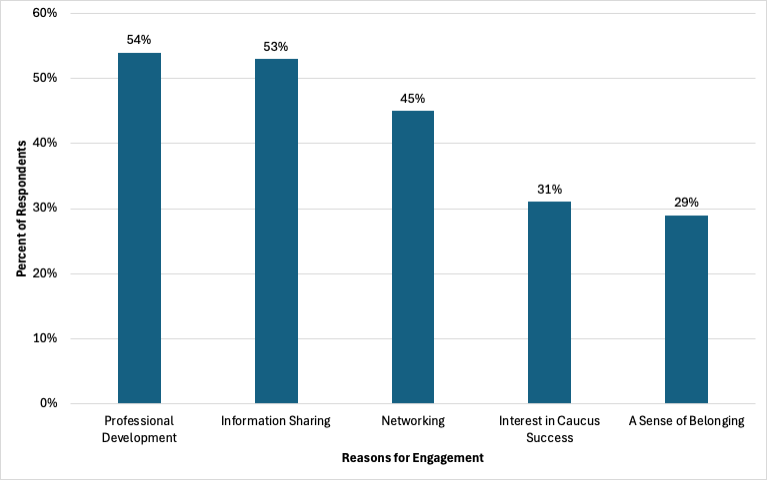
Top five reasons for engagement.

For the 284 respondents who engaged with at least one caucus, the most common way to engage was by reading emails or posts from the listserv (73%, n=206). The second most common way was to attend caucus meetings (55%, n=156), followed by posting or replying to the listserv (51%, n=145), and attending annual or mid-year caucus business meetings (50%, n=142). Outside of participating through the listserv or a variety of caucus meetings, many members (40%, n=113) engaged through attending caucus sponsored events. In addition to the ways members engaged with a caucus, the top 5 reasons for engagement with caucuses are displayed in Figure 2.

Respondents who identified as active members of a caucus were also asked if they felt a sense of belonging to that group. Feelings of belonging varied by caucus, ranging from 0% to 100% of active members. Of the 284 survey respondents who indicated they were an active member of at least one caucus, 77% (n=218) felt a sense of belonging to one or more of their caucuses. Appendix C provides a full overview of involvement and sense of belonging for all caucuses as well as member size, and annual reported activities.

When asked what barriers were experienced to limit the ability to engage in a caucus, the top response was lack of time (80%, n=251/314), which included respondents who felt they were receiving too many emails. Limited benefits and support was also a common barrier (28%, n=87/314), followed by website/caucus pages being too hard to navigate or out of date (20%, n=64/314). Limited benefits and support included respondents who did not see the benefits of joining caucuses, did not have employer support to be involved, and who felt a lack of in-person opportunities were a barrier.

Lack of time was the most common barrier to engaging in an MLA caucus regardless of the frequency of engagement (Table 2). The second most common barrier varied with frequency of engagement, but included limited benefits and support, difficulties navigating the website, lack of clarity on leadership expectations and opportunities, and lack of belonging.

**Table 2:** Frequency of engagement and barriers experienced.

	Daily	Weekly	Monthly	Quarterly	Annually	Never
No Barriers Experienced	4	12	8	3	1	2
Cost of MLA Membership	1	10	12	3	1	3
Difficulty Navigating Webpages	6	19	19	14	2	4
Too Many Caucuses	4	21	13	9	2	3
Lack of Time	24	93	69	37	11	17
Lack of Belonging	6	17	14	4	3	6
Limited Benefits and Support	6	20	18	15	10	18
Leadership Expectations and Opportunities	2	17	20	10	3	4
Lack of Awareness	2	10	12	9	4	7

Despite these barriers, the majority (63%, n=195/312) of respondents planned to remain an active caucus member, with 46% (n=142/312) planning to recommend caucuses to colleagues. Additionally, many respondents planned to volunteer for other communities within MLA (43%, n=133/312) and encourage others to participate in caucus activities (42%, n=132/312), while 22% (n=70/312) planned to volunteer for a caucus leadership position within the next 2-3 years. A full list of engagement activities, reasons for engagement, top barriers to engagement and future plans for engagement can be found in Appendix E.

### Sense of Belonging to the MLA Organization from Survey

When asked to respond to the statement ‘I feel a sense of belonging in MLA’, 61% (n=192/316) of overall respondents either agreed or strongly agreed that they feel a sense of belonging. In contrast, 12% (n=39/316) disagreed or strongly disagreed. A large portion (27%, n=85/316) responded that they were neutral on this statement. Similarly, of the 42 solo librarians who responded to the survey, 60% (n=25) agreed that they felt a sense of belonging in MLA, 12% (n=5) disagreed, and 29% (n=12) were neutral.

Table 3 illustrates the number of caucuses a respondent joined with breakouts by their reported sense of belonging in the MLA organization as a whole. Of the 104 respondents who were in 3-5 caucuses, 70 strongly agreed or agreed that they felt a sense of belonging, followed by 43 of the 56 respondents who were in 9-19 caucuses.

**Table 3:** Number of caucuses joined and sense of belonging in MLA.

	Strongly Disagree	Disagree	Neither Agree nor Disagree	Agree	Strongly Agree
None	1	5	16	7	2
1-2	2	9	20	27	7
3-5	2	11	21	58	12
6-8	0	3	13	23	8
9-19	2	0	11	29	14
20+	3	1	3	3	2

### Reported Activities from Thematic Analysis

While ten activity types were identified through the thematic analysis, caucuses reported a median number of three activity types each year, with a range from zero to eight. The most commonly reported activity type was member engagement in subgroup work such as working groups, task forces, or committee efforts, with a median of twenty-nine caucuses each year. Tied for the next most common activities type, with a median of twenty-four caucuses each year, was hosting an Experience MLA event or business meetings each year. A median of twenty caucuses reported hosting a webinar or discussion event each year, while sixteen reported hosting a collaborative event or sponsored content at the MLA annual meeting. With the lowest reporting rate, a median of only three caucuses each year reportedly sought out in-person opportunities. The full list of activity types reported in caucus annual reports can be found in Appendix B.

Table 4 reports the top 5 caucuses organized by the reported sense of belonging. In each of these caucuses, at least 80% of respondents who reported that they were an active member of the caucus felt a sense of belonging to the caucus. The median number of activity types reported by the caucus and the total number of members for each caucus are also reported in Table 4 highlighting the top 5 caucuses’ according to reported sense of belonging.

**Table 4:** Caucus involvement and belonging highlights.

Caucus Name	Reported Sense of Belonging*	Count of Annually Reported Activity Types**	Member Size***
Vision Science	100%	5	59
New Members	88%	3	800
Public Services	83%	4	215
Hospital Library	82%	5.5	594
Animal and Veterinary Information Specialist	80%	6	124

* Reported as percent of reported active

** Reported as median number of activity types offered per year

** Member size retrieved on October 4, 2023

Appendix C provides a full overview of involvement and belonging for all caucuses as well as member size, annually reported activities, and survey results.

## DISCUSSION

The results of this survey provide insight into the factors impacting member engagement and commitment to MLA Caucuses. In terms of member engagement with MLA Caucuses, the top reasons for engagement aligned with the reported reasons people join professional library organizations in general, namely professional development, information sharing, and networking [7, 8]. The majority of engagement activity occurred through caucus listservs followed by attendance at caucus meetings and sponsored events. Although a combined 58% of respondents engaged with caucuses either weekly or monthly, 2% of members who were in at least one caucus never engaged with them. These findings underscore the importance of utilizing caucus listservs to communicate targeted and relevant information, and for caucuses to schedule meetings and events, such as webinars, discussions, networking sessions, business meetings, or content sessions during annual meetings.

In alignment with previous findings [3,4], lack of time was the biggest barrier for all respondents This is not surprising, as previous studies [11,12] have shown that academic librarians experience role overload and increasing demands on their time as they are asked to do more with less. Tenure track librarians in particular experience additional stress related to expectations for research and service [13].

The second most common barrier was limited benefits and support, which included respondents who did not see the benefits of joining caucuses, did not have employer support to be involved, and who felt a lack of in-person opportunities. Limited benefits and support may also impact members who feel that the cost of MLA membership is too high, especially for members who do not have employer support to be involved.

Rounding out the top three barriers was the perceived difficulty of website and caucus page navigation, and the concern that content was out of date, which echoes results and recommendations from previous MLA findings [3,4]. This barrier is very closely related to lack of awareness of how to join or engage with caucuses, and the lack of understanding of leadership expectations and opportunities. While some of these barriers can be improved through guidance from caucus leadership, the organization website and caucus page navigation will require coordination with MLA organizational leadership. It is important to note that this survey was conducted six months prior to the launch of the new MLA site redesign in summer 2024. There also remains a need for increased awareness about the different caucuses, including how to join them and how to get involved, as increasing awareness may also increase the perceived benefit of caucuses.

Despite these barriers, the overall future plans for engagement in the survey were positive. This is important because past research has shown that engagement significantly explains commitment to an organization [9]. Most respondents planned to remain active with MLA caucuses in some form, and many of the survey respondents planned to recommend caucuses to colleagues, volunteer for other communities within MLA, and encourage participation in caucus activities. However, only 22% of respondents planned to volunteer for a caucus leadership position in the next 2-3 years. This may indicate the impact that barriers such as lack of time, unclear leadership expectations and opportunities, and limited benefits and support could be having on engagement.

Although the majority of survey respondents stated that they have either currently, or previously, held a leadership role, nearly half of all respondents indicated that they had not held any leadership positions since the transition to caucuses. This finding may be due to the high percentage of new members (23%) who responded but may also indicate the difficulty that these members have in identifying leadership expectations and opportunities. Given the large number of caucuses and the relatively low percentage of respondents who planned to volunteer for a caucus leadership position in the future, leadership development of current MLA members may be needed to keep caucuses sustainable.

While 40+ caucuses may seem like a large number of caucuses for members to join, each caucus serves a different function and meets the needs of different user groups. The number of available caucuses may actually increase member engagement and belonging if the variety provides more options for members to find a caucus of interest. This is supported by the 69% of respondents who felt a sense of belonging to one or more of their caucuses. Also of interest, the size of the caucus, or overall number of caucus members, did not seem to correspond with sense of belonging. This was demonstrated in both Table 4 highlights and the full data available in Appendix C.

It's important to note that the thematic review of annual reports showed that several caucuses have disbanded since 2019 due to waning interest or merger with another caucus with similar populations and functions. These included all caucuses that were reporting only one activity type per year. These mergers demonstrate a healthy fluctuation of member interests and consolidation of efforts allowing for increased engagement, activities, and membership.

A major limitation identified during the thematic analysis portion of this study was the difficulty of tracking what more than forty caucuses were doing. There seemed to be little to no standardization, guidelines, or support in annual reporting for caucus leadership and current chairs may have only had access to previously submitted reports for their own caucus as guidance. This led to a wide variation in what was reported and no information was reported regarding the rationale for why certain activities were selected over others For example, the authors expected to find that all caucuses were hosting at least one business meeting open to participation from all members, as this is required by MLA, but it was very difficult to uncover if and when those meetings took place and what they looked like. Though the ability to hold a wide variety of activities is a strength of the caucus structure and there is no one size fits all template, caucuses could benefit from additional guidance and a more structured reporting template so that members can have a better understanding of what each caucus is currently doing.

Another limitation of this study was the survey response rate and restriction of the data analysis to descriptive statistics. The low response rate compared to the total membership means the results might not fully represent the entire group. Our survey was distributed in late November and early December 2023 and collected 317 responses, for an estimated 13% response rate. This is lower than previously reported MLA engagement surveys which reported a 25% response rate from a January - February 2017 member survey [5] and a 34% response rate from October 2019 [6]. Due to the limited time frame of the project, the authors were unable to conduct inferential testing on this data which may limit the generalizability of results. Additionally, because the survey was comprised primarily of Likert style questions rather than free text responses, this study does not include a qualitative component exploring the rationale and affective feelings behind participants’ responses.

## PRELIMINARY RECOMMENDATIONS

The goal of this study was to answer the question “What factors influence member engagement and commitment to an MLA caucus?” Based on these findings, the authors propose six preliminary recommendations to enhance leadership recruitment and member engagement with MLA caucuses. Recommendation one is drawn directly from the survey results. Recommendations two through six are drawn from the author's experience of analyzing the survey, conducting the thematic analysis of the annual reports, and visiting Community Council. The authors hope that these recommendations will be read and considered by the general membership of MLA as well as by MLA leaders and staff.

### Recommendation One: Focus Caucus Activities Around the Top Reasons for Engagement

Caucus leadership should focus caucus activities around the top reasons that members engage with caucuses, such as professional development, information sharing, and networking. While annual reports show that many caucuses are already engaging in these activities, caucus leaders should consider surveying their membership about which specific types of professional development, information sharing, and networking activities may be of interest.

### Recommendation Two: Create Caucus Specific Guidance Documents for Incoming Leaders

Caucus chairs should create caucus specific leadership guidelines to address the reported barrier of lack of clarity on leadership expectations and opportunities. These could include the responsibilities of past-chair, chair, and chair elect; deadlines for required documentation such as reports and nomination slates; how to request an MLA sponsored Zoom link; popular activity types with general descriptions, dates held, and historical participation numbers as well as brief charters, goals, and/or deliverables from standing subgroups and working groups.

### Recommendation Three: Create Separate Leadership Introduction Meeting and Guidance Document for MLA Caucus Leaders

To address a lack of clarity surrounding leadership expectations it would be beneficial to have a guidance document outlining the reporting requirements and deadlines for MLA caucuses that is easily accessible for all MLA members. A template or suggested guidelines could be produced or maintained by the MLA Community Council to which all caucuses formally report. Additionally, a leadership introduction meeting for caucuses should be held separately from the leadership introduction meeting for committees and juries. MLA caucuses serve a different function than committees and juries, and caucus leaders would benefit from a leadership introduction tailored to the unique needs of caucuses such as how to host events and engage members.

### Recommendation Four: Standardized Annual Reporting

Preliminary recommendations following this project are to standardize the caucus annual report form and make the final reports more transparent and easier to find. A task force could be appointed by the MLA Community Council to revise the current annual reporting form to include guidance about the types of information that should be included in each section. The task force could also investigate ways to make the information from the annual reports more transparent. Currently, the annual reports are only available as a single PDF document. It could be beneficial to create an interactive dashboard highlighting information from each caucus. Following the MLA website redesign, it is important for MLA staff and leadership to continue to address the difficulties experienced when navigating the website and caucus pages.

### Recommendation Five: Use Community Council as a Discussion Forum

As the representative body of Caucus leaders, Community Council can provide a forum for caucus leaders to discuss what has been working for caucus engagement, rather than as a recap of what can be found in annual/mid-year reports. Alternatively, Community Council can meet once a quarter instead of biannually, allowing for two meetings for reviewing reports and two meetings for active discussion and action items. This would allow more time for caucus leaders to share ideas and strategize specific activities and efforts aimed to increase member engagement with both individual caucuses and through caucus collaboration.

### Recommendation Six: Reinstate Experience MLA

Experience MLA was a popular program held from 2021 - 2023 that provided an opportunity for increased caucus engagement and networking, including no-cost activities and free MLA trial memberships. Over half of all caucuses emphasized their participation in Experience MLA, and annual reports from 2021 - 2023 showed that hosting an Experience MLA event was tied with business meetings for the second most common activity type. Though Experience MLA was initially focused on recruiting new members to MLA, it also allowed current MLA members to learn more about the different caucuses without being required to join the caucus. Experience MLA was not held in 2024 or 2025 which meant that there were less opportunities for general MLA members to engage with a variety of caucuses. The opportunity to participate in a variety of caucus events during this time may have provided additional value to existing members and may have increased engagement and retention of current caucus members.

## CONCLUSION

Increasing member engagement and commitment to MLA caucuses, as well as reducing barriers for new and existing members, will require a joint effort from caucus leaders, MLA Community Council, and MLA staff. Individual caucus leaders can focus on creating caucus specific guidance documents and hosting activities around the top reasons for engagement. MLA Community Council will need to work with MLA staff to oversee systemic changes such as standardizing the annual reporting form, creating a guidance document for caucus leaders, reinstating Experience MLA, and addressing issues with navigating the MLA website and caucus pages. To foster sustainable engagement and commitment within MLA caucuses, members must find value in their participation, highlighting the importance of embracing that together, we are MLA.

## Data Availability

Data associated with this article are available in the Open Science Framework at https://osf.io/7umfd/

## SUPPLEMENTAL FILES

**Appendix A:** Survey Instrument

**Appendix B:** Reported Activities by Caucuses

**Appendix C:** Demographic Responses

**Appendix D:** Caucus Engagement
