# Supplementary material for: Revisiting we are MLA: an exploration of member engagement and commitment with the Medical Library Association's caucuses
Source: J Med Libr Assoc. 2026 Feb 17;114(1):11–20. doi: 10.5195/jmla.2026.2183 (PMC12947922; doi:10.5195/jmla.2026.2183)
Supplement: Supplementary file 1 — Appendix A: Survey Instrument [file jmla-114-1-11-s01.docx]

**Appendix A.** Survey Instrument

**Assessment of Caucus Engagement in the Medical Library Association**

The [BLINDED] MLA Rising Stars cohort have been tasked with exploring how participants engage with caucuses and to discover perceived barriers to engagement, with the ultimate goal of increasing sustainability and engagement of the MLA caucuses. Specifically, our study addresses the following question: What factors influence member engagement and commitment to an MLA caucus?

This online survey should take approximately 5-10 minutes to complete and will close December 15th. Please view the informed consent statement below. If you have questions about the survey or the procedures, you may contact BLINDED.

Informed Consent Statement

You are being asked to participate in this research study because you are currently a member of the Medical Library Association (MLA). Participation in this research is voluntary.

Participating in this study involves filling out an online survey in Redcap consisting of multiple-choice questions. This online survey should take approximately 5-10 minutes to complete and will close in 2 weeks.

Responses are anonymous. Submission data (IP addresses, browsers, and other personal identifiers) are not being collected. The results may be published, but no one will be able to identify you as a participant.

There are no direct benefits to you from participating in this survey, however your participation may help us learn more about how participants engage with caucuses and discover perceived barriers to engagement. This information may lead to changes in the MLA caucus organization that may benefit future members.

Preliminary results from this survey will be released at the MLA annual conference in May 2024. Your participation is voluntary, and you may decide not to participate in the survey and may exit the survey at any time without penalty. You are free to decline to answer any questions you do not want to answer for any reason. If you have questions about the survey or the procedures, you may contact BLINDED

By continuing to the survey, you are choosing to participate in the survey, which implies your understanding and willingness to participate.

Demographic Questions

**1. In which type of setting do you work?**

**[SELECT ONE]**

- Academic Library (2 year, 4 year, graduate, or postgraduate)
- Corporate (e.g. Insurance, Pharmaceutical, Publishing)
- Federal or State Library (non-academic)
- Hospital/Healthcare System Library
- Public Library
- Student (part time or full time)
- Unemployed
- Other (please specify):

**2. [Branching- from #1] Do you currently consider yourself a solo-librarian?**

**[SELECT ONE]**

- Yes
- No, but I have been a solo-librarian in the past
- No, I am employed as part of a team of librarians
- No, I am not currently employed as a librarian

**3. In which country do you currently live and work or go to school? ^(Pionke)^**

**[SELECT ONE]**

- United States
- Canada
- Outside the United States or Canada

**4. Please indicate the racial OR ethnic groups with which you identify:**

**[SELECT ALL THAT APPLY]**

- Prefer not to respond
- Asian or Asian American
- Black or African American
- Hispanic/Latinx
- Middle Eastern/North African
- Native American or Alaskan Native
- Native Hawaiian or Pacific Islander
- White or Caucasian
- Multiracial
- Other (please specify):

**5. What is your current age (range)? ^(Pionke)^**

**[SELECT ONE]**

- Prefer not to respond
- 20-29 years
- 30-39 years
- 40-49 years
- 50-59 years
- 60–64 years
- 65-70 years
- 71-75 years
- 76 years or more

MLA Specific Questions

**6. Are you a current member of the Medical Library Association (MLA)? ^(Pionke)^**

**[SELECT ONE]**

- Yes
- No

7. **How many years have you been an MLA member? ^(Pionke- EDITED)^**

**[SELECT ONE]**

- Less than 1
- 1-2
- 3-4
- 5-9
- 10-14
- 15-19
- 20-24
- 25+

**8. Does your employer pay for your annual MLA membership? ^(Pionke)^**

**[SELECT ONE]**

- Yes, full membership fee outside of any professional development funds
- Yes, full membership fee if I choose to use professional development for membership
- Yes, part of my membership (e.g. offers the $50 rebate option)
- No
- Other (please specify):

**9. Since 2019, have you held (or do you currently hold) a leadership position in any MLA group or community (e.g. caucus, committee, jury, etc)?**

**[SELECT ONE]**

- Yes, I currently hold a leadership position
- Yes, I previously held a leadership position anytime between 2019-2023
- No, I did not hold a leadership position anytime between 2019-present

**10. [Branching from #9] In which MLA member community do you hold (or have you previously held) a leadership position:**

**[SELECT ALL THAT APPLY]**

- MLA Board of Directors
- Standing Committees
- Juries
- Editorial Boards
- Taskforces
- Community Council
- Chapter Council
- Caucuses
- Domain Hubs
- Other (please specify):

**11. Other than a caucus, have you been (or are you currently) a member of an MLA community since 2019? (select all that apply).**

**[SELECT ALL THAT APPLY]**

- None
- MLA Board of Directors
- Standing Committees
- Juries
- Editorial Boards
- Taskforces
- Community Council
- Chapter Council
- Domain Hubs
- Other (please specify):

Participatory Behavior in MLA Caucuses Questions

**12. How often do you feel you engage [in any way] with an MLA caucus?**

**[SELECT ONE]**

- Daily
- Weekly
- Monthly
- Quarterly
- Annually
- Never

**13. About how many MLA caucuses have you currently joined? [SELECT ONE]**

- None
- 1-2
- 3-5
- 6-8
- 9-19
- 20+

**14. [Branching from #13] In which MLA caucus or caucuses do you consider yourself to be an active member?**

**[SELECT ALL THAT APPLY]**

- I am a member of MLA caucuses but do not consider myself active
- Academic Librarians
- Accessibility and Disability
- African American Medical Librarians Alliance
- Animal and Veterinary Information Specialist
- Basic Science
- Cancer Librarians
- Clinical Librarians and Evidence Based Practice
- Collection Development
- Complementary and Integrative Health
- Consumer and Patient Health Information Services
- Data
- Dental
- Federal Libraries
- Health Association and Corporate Librarians
- Health Humanities
- History of the Health Sciences
- Hospital Library
- International Cooperation
- Interprofessional Education and Practice
- Latinx
- Leadership and Management
- LGBTQIA+
- Libraries in Health Sciences Curriculums
- Medical Informatics
- Medical Library Education
- New Members
- Nursing and Allied Health Resources and Services
- Osteopathic Libraries
- Pediatric Librarians
- Pharmacy and Drug Information
- Public Health/Health Administration
- Public Services
- Research
- Resource Sharing
- Scholarly Communications
- Social Justice and Health Disparities
- Systematic Reviews
- Technical Services
- Technology in Education
- Translational Sciences Collaboration
- User Experience
- Vision Science
- Other (please specify)

**15. [Branching from #14] For the caucuses in which you consider yourself an active member, in what ways do you engage with that caucus?**

**[SELECT ALL THAT APPLY]**

- Reading the listserv (emails or posts)
- Posting or replying to the listserv (emails or posts)
- Attending caucus business meetings
- Attending caucus meetings (monthly, quarterly, non-business meetings, etc.)
- Attending caucus events (ex: Academic Librarians Caucus Writing Accountability Group Drop-In)
- Attending the caucus/community luncheon at the MLA annual meeting
- Partnering with caucus members on projects or presentations
- Volunteering for formal caucus leadership (domain hub rep, chair elect, chair, or other elected position)
- Volunteering for informal caucus leadership (hosting, planning, sub-committees, or other non-elected position)
- Other (please specify):

**16. [Branching from #14] Why do you choose to engage with the caucuses in which you consider yourself to be an active member?**

**[SELECT ALL THAT APPLY]**

- Networking
- Professional development
- Leadership opportunities
- Information sharing - I want to ask for guidance and/or collaborate with others
- I feel a sense of belonging
- I want to see my caucus be successful
- I like to volunteer/participate
- I have friends or colleagues (or frolleagues) active in the caucus
- I am encouraged to be involved by my organization/mentor
- I need to show involvement for promotion and/or tenure purposes
- I need Academy of Health Information Professionals (AHIP) points
- Other

**17. [Branching from #14] Of the caucus(es) you consider yourself active in, to which do you have the greatest sense of belonging?**

**[SELECT ALL THAT APPLY]**

- None
- Academic Librarians
- Accessibility and Disability
- African American Medical Librarians Alliance
- Animal and Veterinary Information Specialist
- Basic Science
- Cancer Librarians
- Clinical Librarians and Evidence Based Practice
- Collection Development
- Complementary and Integrative Health
- Consumer and Patient Health Information Services
- Data
- Dental
- Federal Libraries
- Health Association and Corporate Librarians
- Health Humanities
- History of the Health Sciences
- Hospital Library
- International Cooperation
- Interprofessional Education and Practice
- Latinx
- Leadership and Management
- LGBTQIA+
- Libraries in Health Sciences Curriculums
- Medical Informatics
- Medical Library Education
- New Members
- Nursing and Allied Health Resources and Services
- Osteopathic Libraries
- Pediatric Librarians
- Pharmacy and Drug Information
- Public Health/Health Administration
- Public Services
- Research
- Resource Sharing
- Scholarly Communications
- Social Justice and Health Disparities
- Systematic Reviews
- Technical Services
- Technology in Education
- Translational Sciences Collaboration
- User Experience
- Vision Science
- Other (please specify)

**18. What barriers do you experience that limit your ability to engage in a caucus?**

**[SELECT ALL THAT APPLY]**

- Unaware of MLA Caucuses
- Lack of Time
- I don't see the benefits
- Not sure where to start, don’t know how to get involved
- Caucus leadership expectations/requirements are unclear
- Leadership roles already filled/don’t want to compete to be elected
- Too many caucuses
- I don't feel like I belong
- I don't have employer support
- Too many emails
- Website/Caucus pages are too hard to navigate or out of date
- No in-person opportunities to meet
- None, no barriers experienced
- Other (please specify):

Wrap Up Questions

**19. In the next 2-3 years, I am likely to**

**[SELECT ALL THAT APPLY]**

- Encourage others to participate in caucus activities
- Recommend caucuses to anyone who wants to get involved with MLA
- Continue to be an active member of one or more caucuses
- Volunteer for caucus leadership
- Volunteer for another MLA community (jury, committee, etc.)

**20. I feel a sense of belonging in MLA ^(Pionke- EDITED)^**

**[LIKERT SCALE]**

1 (strongly disagree) to 5 (strongly agree)
