# Supplementary material for: Revisiting we are MLA: an exploration of member engagement and commitment with the Medical Library Association's caucuses
Source: J Med Libr Assoc. 2026 Feb 17;114(1):11–20. doi: 10.5195/jmla.2026.2183 (PMC12947922; doi:10.5195/jmla.2026.2183)
Supplement: Supplementary file 2 — Appendix B: Reported Activities by Caucuses [file jmla-114-1-11-s02.docx]

**Appendix B.** Number of Caucuses Reporting Each Activity Type by Year

| **Activity Type** | **Number of Caucuses Reporting Activity Type Per Year  Separated By Annual Reporting Year** | | | | **Median Number of Caucuses Reporting Activity Type from 2019 - 2023** |
| --- | --- | --- | --- | --- | --- |
|  | **2019-2020 Annual Report** | **2020-2021 Annual Report** | **2021-2022 Annual Report** | **2022-2023 Annual Report** |  |
| Working Groups, Task Force and/or Committees | 21 | 32 | 34 | 26 | **29** |
| Business Meetings | 13 | 27 | 28 | 21 | **24** |
| Experience MLA Event | N/A | 20 | 25 | 24 | **24** |
| Webinars and/or Discussions | 8 | 18 | 26 | 23 | **20.5** |
| Collaborations with other Caucuses or Associations | 15 | 17 | 23 | 15 | **16** |
| Sponsored Content at Annual Meeting | 13 | 15 | 18 | 17 | **16** |
| Newsletter and/or Member Highlights | 9 | 14 | 22 | 13 | **13.5** |
| Networking Opportunities | 3 | 15 | 14 | 12 | **13** |
| Other Caucus Activities | 4 | 5 | 4 | 3 | **4** |
| In-Person Events | 2 | 1 | 4 | 4 | **3** |
