# Supplementary material for: Revisiting we are MLA: an exploration of member engagement and commitment with the Medical Library Association's caucuses
Source: J Med Libr Assoc. 2026 Feb 17;114(1):11–20. doi: 10.5195/jmla.2026.2183 (PMC12947922; doi:10.5195/jmla.2026.2183)
Supplement: Supplementary file 3 — Appendix C: Demographic Responses [file jmla-114-1-11-s03.docx]

**Appendix C.** Demographic Responses

| **Work Setting** | **% (n=317)** |
| --- | --- |
| Academic (2-year, 4-year, graduate, or postgraduate) | 63% |
| Association, non-profit, or trust | 2% |
| Corporate (e.g. insurance, pharmaceutical, publishing) | 2% |
| Federal or state (non-academic) | 2% |
| Hospital/healthcare system | 24% |
| Public (non-academic) | 1% |
| Retired | 2% |
| Student (part-time or full-time) | 1% |
| Unemployed | 3% |

| **Solo Librarian** | **% (n=303)** |
| --- | --- |
| Yes | 14% |
| No, but I have been a solo librarian in the past | 18% |
| No, I am employed as part of a team of librarians | 62% |
| No, I am not currently employed as a librarian | 6% |

| **Race or Ethnicity** | **% (n=310)** |
| --- | --- |
| Asian or Asian American | 3% |
| Black or African American | 6% |
| Hispanic/Latinx | 3% |
| Multiracial | 4% |
| Native American or Alaskan Native | 1% |
| White or Caucasian | 72% |
| Prefer not to respond | 8% |
| Chose not to answer | 3% |

| **Country of Residence** | **% (n=315)** |
| --- | --- |
| United States | 96% |
| Canada | 2% |
| Outside the United States or Canada | 2% |

| **Age** | **% (n=315)** |
| --- | --- |
| 20-29 | 3% |
| 30-39 | 19% |
| 40-49 | 27% |
| 50-59 | 27% |
| 60-64 | 10% |
| 65-70 | 4% |
| 71-75 | 4% |
| 76+ | 1% |
| Prefer not to respond | 5% |
