# Supplementary material for: Revisiting we are MLA: an exploration of member engagement and commitment with the Medical Library Association's caucuses
Source: J Med Libr Assoc. 2026 Feb 17;114(1):11–20. doi: 10.5195/jmla.2026.2183 (PMC12947922; doi:10.5195/jmla.2026.2183)
Supplement: Supplementary file 4 — Appendix D: Caucus Engagement [file jmla-114-1-11-s04.docx]

**Appendix D.** Caucus Engagement

| **Questions** | **Responses^1^ (n=317)** |
| --- | --- |
| **Ways of Engagement**   - Reading the listserv (emails or posts) - Posting or replying to the listserv (emails or posts) - Attending annual or mid-year caucus business meetings - Attending caucus meetings (monthly, quarterly, non-business meetings, etc.) - Attending caucus events (ex: Academic Librarians Caucus Writing Accountability Group Drop-In) - Attending the caucus/community luncheon at the MLA annual meeting - Partnering with caucus members on projects or presentations - Volunteering for formal caucus leadership (domain hub rep, chair elect, chair, or other appointed or elected position) - Volunteering for informal caucus leadership (hosting, planning, sub-committees, or other non-elected position) - Other (please specify): | 206 (65)  145 (46)  142 (45)  156 (49)  113 (36)  76 (24)  94 (30)  91 (29)  59 (19)  2 (1) |
| **Reason for Engagement**   - Networking - Professional Development - Leadership Opportunities - Information Sharing - I want to ask for guidance and/or collaborate with others - I feel a sense of belonging - I want to see my caucus be successful - I like to volunteer/participate - I have friends or colleagues (or frolleagues) active in the caucus - I am encouraged to be involved by my organization/mentor - I need to show involvement for promotion and/or tenure purposes - I need Academy of Health Information Professionals (AHIP) points - Other (please specify): | 144 (45)  171 (54)  69 (22)  167 (53)  93 (29)  97 (31)  63 (20)  61(19)  25 (8)  43 (14)  39 (12)  6 (2) |
| **Top Barriers to Engagement**   - Lack of Awareness^2^ - Lack of Time^3^ - Real World Applicability^4^ - Lack of Belonging^5^ - Leadership Expectations and Opportunities^6^ - Too many caucuses - Website/caucus pages are too hard to navigate or out of date - Cost of MLA membership - No Barriers Experienced | 44 (14)  251 (79)  87 (27)  50 (16)  56 (18)  52 (16)  64 (20)  30 (9)  30 (9) |
| **Ways will Engage in the Future**   - Encourage others to participate in caucus activities - Recommend caucuses to anyone who wants to get involved with MLA - Continue to be an active member of one or more caucuses - Volunteer for caucus leadership - Volunteer for another MLA community (jury, committee, etc.) - None of the above | 132 (42)  142 (45)  195 (62)  70 (22)  133 (42)  49 (15) |

^1^ Listed as n (%) unless otherwise stated. ^2^ Includes the responses for “Unaware of MLA Caucuses”, “I am not sure how to join one”. and “I don't know how to get involved once I have joined”. ^3^ Includes the responses for “Lack of time” and “Too many emails”. ^4^ Includes the response for “I don’t see the benefits”, “I don’t have employer support, and “No in-person opportunities to meet”. ^5^ Includes the responses for “I don’t feel like I belong” and “I don’t want to ask a silly question”. ^6^ Includes the responses for “Caucus leadership expectations/requirements are unclear” and “Leadership roles already filled/dont want to compete to be elected”
